# Ecological and cultural factors underlying the global distribution of prejudice

**DOI:** 10.1371/journal.pone.0221953

**Published:** 2019-09-06

**Authors:** Joshua Conrad Jackson, Marieke van Egmond, Virginia K. Choi, Carol R. Ember, Jamin Halberstadt, Jovana Balanovic, Inger N. Basker, Klaus Boehnke, Noemi Buki, Ronald Fischer, Marta Fulop, Ashley Fulmer, Astrid C. Homan, Gerben A. van Kleef, Loes Kreemers, Vidar Schei, Erna Szabo, Colleen Ward, Michele J. Gelfand

**Affiliations:** 1 University of North Carolina, Department of Psychology and Neuroscience, Chapel Hill, United States of America; 2 University of Hagen, Institute of Psychology, Hagen, Germany; 3 University of Maryland, Department of Psychology, College Park, United States of America; 4 Yale University, Human Relations Area Files, New Haven, United States of America; 5 University of Otago, Department of Psychology, Dunedin, New Zealand; 6 Victoria University of Wellington, School of Psychology, Wellington, New Zealand; 7 NHH Norwegian School of Economics, Department of Strategy and Management, Bergen, Norway; 8 Jacobs University, Bremen International Graduate School of Social Sciences, Bremen, Germany; 9 National Research University Higher School of Economics, International Laboratory for Sociocultural Research, Moscow, Russia; 10 Hungarian Academy of Sciences, Institute for Cognitive Neuroscience and Psychology, Budapest, Hungary; 11 Eötvös Loránd University, Faculty of Psychology and Education, Budapest, Hungary; 12 Georgia State University, Robinson College of Business, Atlanta, United States of America; 13 University of Amsterdam, Department of Psychology, Amsterdam, The Netherlands; 14 Johannes Kepler University Linz, Department of International Management, Linz, Austria; Sogang University (South Korea), REPUBLIC OF KOREA

## Abstract

Prejudiced attitudes and political nationalism vary widely around the world, but there has been little research on what predicts this variation. Here we examine the ecological and cultural factors underlying the worldwide distribution of prejudice. We suggest that cultures grow more prejudiced when they tighten cultural norms in response to destabilizing ecological threats. A set of seven archival analyses, surveys, and experiments (∑*N* = 3,986,402) find that nations, American states, and pre-industrial societies with tighter cultural norms show the most prejudice based on skin color, religion, nationality, and sexuality, and that tightness predicts why prejudice is often highest in areas of the world with histories of ecological threat. People’s support for cultural tightness also mediates the link between perceived ecological threat and intentions to vote for nationalist politicians. Results replicate when controlling for economic development, inequality, conservatism, residential mobility, and shared cultural heritage. These findings offer a cultural evolutionary perspective on prejudice, with implications for immigration, intercultural conflict, and radicalization.

## Introduction

Recent years have seen a rise of nationalist political parties and policies in many Western nations. In the United States, Donald Trump was elected president in 2016 and has since drafted laws aimed at more aggressive deportation and the construction of a border wall. That same year in Europe, support for populist parties and distrust of ethnic minorities and immigrants rose to a 30-year high [1], resulting in the election of nationalist leaders in Poland and the Czech Republic and potentially contributing to the United Kingdom’s decision to leave the European Union. This escalation of nationalism puzzles academics and policy-makers alike, prompting open questions about the cultural and societal factors that predict prejudice and nationalism.

These geopolitical events expose a troubling lack of research on cultural variation in prejudice and nationalism. Laboratory studies have examined the individual-level cognitive mechanisms behind racial [2–3], religious [4–5], ethnic [6–7], and linguistic [8–9] prejudice, and qualitative studies have examined prejudice within single nations or during critical periods of history [10–11], but few studies have quantitatively examined cross-cultural variation in prejudice using ecological variables (for exceptions, see [12–15]).

This lack of quantitative cross-cultural research partly stems from the fact that most social science studies recruit subjects from the United States and other western, democratic, and highly educated nations [16], leaving prejudice’s tremendous geographic and historic variability largely unexplored. Racial hate crimes [17], religious violence [18], intolerance for non-traditional sexual practices [19], and many other forms of intergroup prejudice [20] vary widely around the world. This cross-cultural covariation is not a contemporary phenomenon. Historical indicators of prejudice such as interhousehold sharing and parochial cooperation also show considerable variation across cultures [21–23]. What environmental and cultural factors underlie this cross-cultural variation in prejudice? And could these same factors explain the rise of nationalist politicians?

## Ecological threat, cultural tightness, and variation in prejudice

Here, we outline a broad research program examining whether cross-cultural variability in prejudice is linked to cultural tightness—the strength of a society’s norms and the strictness of its punishments for deviant behavior—and more distally, to the ecological threats that drive tightness [24]. We define ecological threats as factors from the social or natural environment, broadly defined, that threaten societies’ existence. According to past research, cultures tighten in the face of ecological threat [24–26]. During warfare, disease outbreaks, or resource scarcity, societies are faced with increased coordination pressures across diverse populations of unrelated individuals [27–28]. Evolutionary game theory models and behavioral data suggest that cultural tightness emerges following threat because it curbs defection and increases coordination [28–29].

Yet while tightness encourages coordination through increased social regulation, it also decreases openness [24,30]. Across contemporary nations [24] and US states [29], cultural tightness is negatively correlated with openness to new ideas and societal innovations that disrupt the social order [31–32] We theorize that cultural tightness may also increase prejudice against individuals who are seen as disrupting the social order simply because they practice a minority religion, belong to a minority ethnic group, or have a minority sexual orientation.

Our theory makes two predictions that we test with seven empirical studies. First, we predict that cultural variation in tightness should predict cultural variation in prejudice. Our first 4 studies test this link across different levels of analysis: first across 25 current-day nations (Study 1), then within a single nation by analyzing the 50 United States (Studies 2–3), then across 47 historical pre-industrial societies (Study 4). Our final 3 studies test whether individual people’s support for cultural tightness can predict their prejudiced attitudes and support for nationalist politicians during elections (Studies 5–7). Indeed, one of the strengths of our research is that we test our predictions using a combination of correlational and experimental methods for the link between cultural tightness and prejudice in a variety of comparative samples, consistently replicating our results across studies.

Our second prediction is that tightness should mediate a link between ecological threat and prejudice. TL theory holds that, during times of threat, groups and individuals gravitate towards cultural tightness because tighter norms can better maintain societal order, stability, and coordination [24–26]. If tightness is linked to prejudice, then threat provides the ecological basis to understand variation in prejudice through its influence on tight norms. Indeed, there is evidence that threats like scarcity and pathogen prevalence are associated with prejudice [32–40]. In this research, we test whether a broad array of threats catalyze tight norms which are in turn linked with multiple forms of prejudice in modern and pre-industrial societies. We then replicate this model at the individual level, testing whether the salience of threats facing society can predict people’s support for cultural tightness, which in turn predicts their prejudiced attitudes.

## Study 1: Prejudice in current-day nations

Study 1 examined the link between cultural tightness and prejudice in current-day nations using data from the six waves of the World Values Survey (WVS). We operationalized prejudice through six questions that asked whether or not participants would be willing to live near (a) people from a different race, (b) people from a different religion, (c) immigrants, (d) people who spoke a different language, (e) people of a different sexual orientation, and (f) unmarried cohabitants. We focused on these questions because they mapped onto Goffman’s list of “tribal stigmas:” aspects of a person that confer a group identity and are frequent targets of prejudice [41]. They also quantified bias towards a number of groups using the exact same language, and the scale showed appropriate reliability (Cronbach’s α = .75), allowing us to average them into a single prejudice index. Our supplementary materials contain several other measures of prejudice which show similar results.

We operationalized nation-level tightness and ecological threat using the score from Gelfand and colleagues [24]. Our ecological threat index is described in detail on p. 12 of Gelfand and colleagues [24]. It contains standardized values of historical population density in the year 1500 [42], food deprivation [43], years of life lost to communicable disease [44], vulnerability to disaster [45], and historical territorial conflicts [24].

In total, 117,157 individuals from 25 countries had data on prejudice, cultural tightness, and ecological threat, allowing for appropriately powered multi-level analyses, wherein intercepts varied randomly across nations. We also controlled for an estimate Gross Domestic Product per Capita at purchasing power parity (GDPPP), inequality (GINI), and WVS wave in order to model the effects of economic development, social inequality, and time. GDPPP and GINI were derived from World Bank estimates [46]. We controlled for these variables (and all other control variables used in this paper) by entering them as predictors into our regressions.

### Results

#### Cultural tightness and prejudice

**Fig 1:**
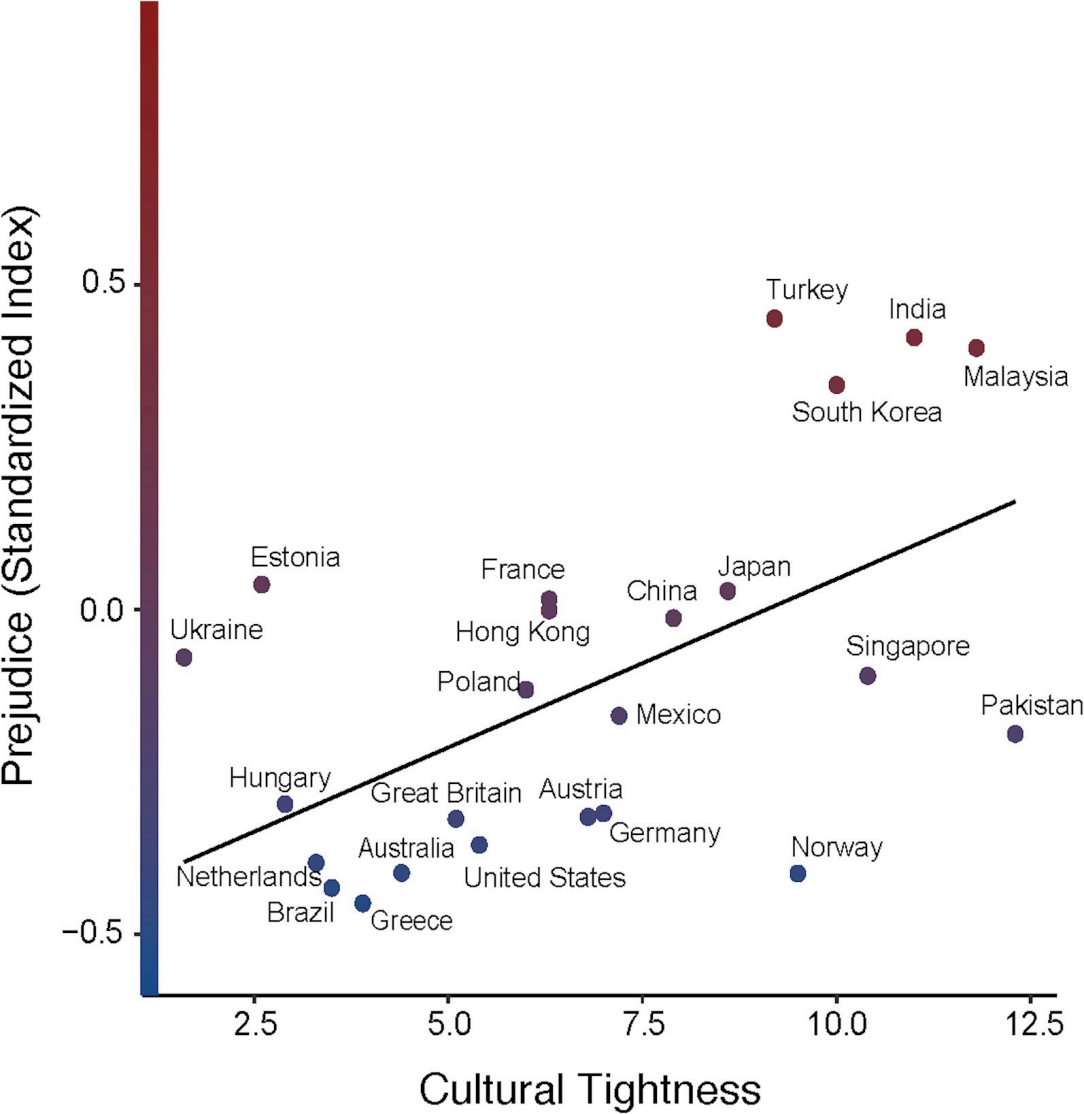
The relationship between cultural tightness and prejudice. Prejudice has been standardized in this figure. Prejudice is operationalized through the refusal to live next to individuals from seven commonly stigmatized groups.

Our first prediction was that tightness would correlate with prejudice. In support of this prediction, nation-level tightness robustly predicted prejudice across nations, *b* = .05, *SE* = .02, *t* = 3.25, *p* = .004, *R*^2^_level 2_ = .30 (see Fig 1), even with our control variables. Indeed, tightness correlated with each indicator of prejudice, predicting prejudice towards people of a different race, *OR* = 1.13, *z* = 2.92, *p* = .004, people of a different religion, *OR* = 1.20, *z* = 3.81, *p* < .001, people who spoke a different language, *OR* = 1.10, *z* = 2.01, *p* = .04, gay people, , *OR* = 1.09, *z* = 2.14, *p* = .03, immigrants, *OR* = 1.18, *z* = 4.17, *p* < .001, and unmarried cohabitants, *OR* = 1.38, *z* = 6.17, *p* < .001.

#### Ecological threat, cultural tightness, and prejudice

Our second prediction was that tightness would mediate the relationship between ecological threat and prejudice. Preliminary analyses showed that ecological threat directly predicted tightness across nations, *b* = .25, *SE* = .08, *t* = 3.23, *p* = .004, *R*^2^_level 2_ = .26, but did not directly predict prejudice, *b* = .008, *SE* = .009, *t* = .91, *p* = .38, *R*^*2*^_level 2_ = .04. Importantly, even when there is no total effect between two variables, there may be an indirect effect through a mediator [47]. The conditions for probing mediation are that (a) the X (threat) variable predicts the M (tightness) variable, and that (b) the M (tightness) variable predicts the Y (prejudice) variable even controlling for the X variable [47]. Since we had already confirmed (a), and a multiple regression confirmed (b), *b* = .06, *SE* = .02, *t* = 2.94, *p* = .009, *R*^2^_level 2_ = .29, we tested for an indirect effect.

Using Monte Carlo simulation, which is well-suited for testing multi-level mediation [48], we confirmed that there was a significant indirect association between ecological threat and prejudice across nations through cultural tightness, 95% *CIs* [.003, .03]. Threat remained a non-significant predictor of prejudice when controlling for cultural tightness, *b* = -.01, *SE* = .009, *t* = -0.75, *p* = .46, *R*^2^_level 2_ < .001. There was no support for an alternative model where prejudice mediated the relationship between threat and tightness, further supporting our theoretical model.

## Study 2: Explicit prejudice across states in the USA

We next tested whether a relationship between cultural tightness and prejudice would replicate across federal states in the US, which have also been differentiated on cultural tightness and ecological threat in past analyses [29]. For example, Harrington and Gelfand [29] found that relatively tighter states such as Alabama and Virginia have higher execution rates, less access to alcohol, and more frequent corporal punishment in schools compared to relatively looser states like Nevada or Washington. Like nations, tighter states have historically faced more threat (e.g., infectious diseases, floods, resource scarcity) than looser states [29]. Study 2 examined whether these tighter states would show more prejudice than looser states, controlling for variation in wealth (income), residential mobility across states, and political conservatism. Controlling for political conservativism was important in Study 2 because tightness and conservatism correlate highly across American states [29].

We gathered data on prejudice using the 1973–2010 waves of the General Social Survey (GSS). To measure prejudice, we used a set of items where participants would indicate whether they would oppose a relative marrying (a) a Black person, (b) a Jewish person, (c) a Hispanic person, (d) an Asian person, and (e) whether participants support gay marriage. Participants responded to each item on a 1–5 scale, where higher values communicated less willingness to allow marriage, and the items formed a reliable prejudice index (Cronbach’s α = .88).

We measured state-level cultural tightness and ecological threat using the scores from Harrington and Gelfand [29]. Harrington and Gelfand’s ecological threat scale includes measures of parasite stress [49], resource scarcity [50], and past conflict [29].

In total, 11,577 individuals from the 50 federal states had data on prejudice. We gathered data on residential mobility via the number of people in the general social survey who reported having moved to a different state than the one in which they had grown up (67% of the sample) and on conservativism via vote-share for Mitt Romney in the 2012 presidential election, which was the year that our tightness data were collected. We used multi-level regression wherein intercepts were modeled as randomly varying across states to test the relationship between cultural tightness and each measure of prejudice.

### Results

#### Cultural tightness and prejudice

**Fig 2:**
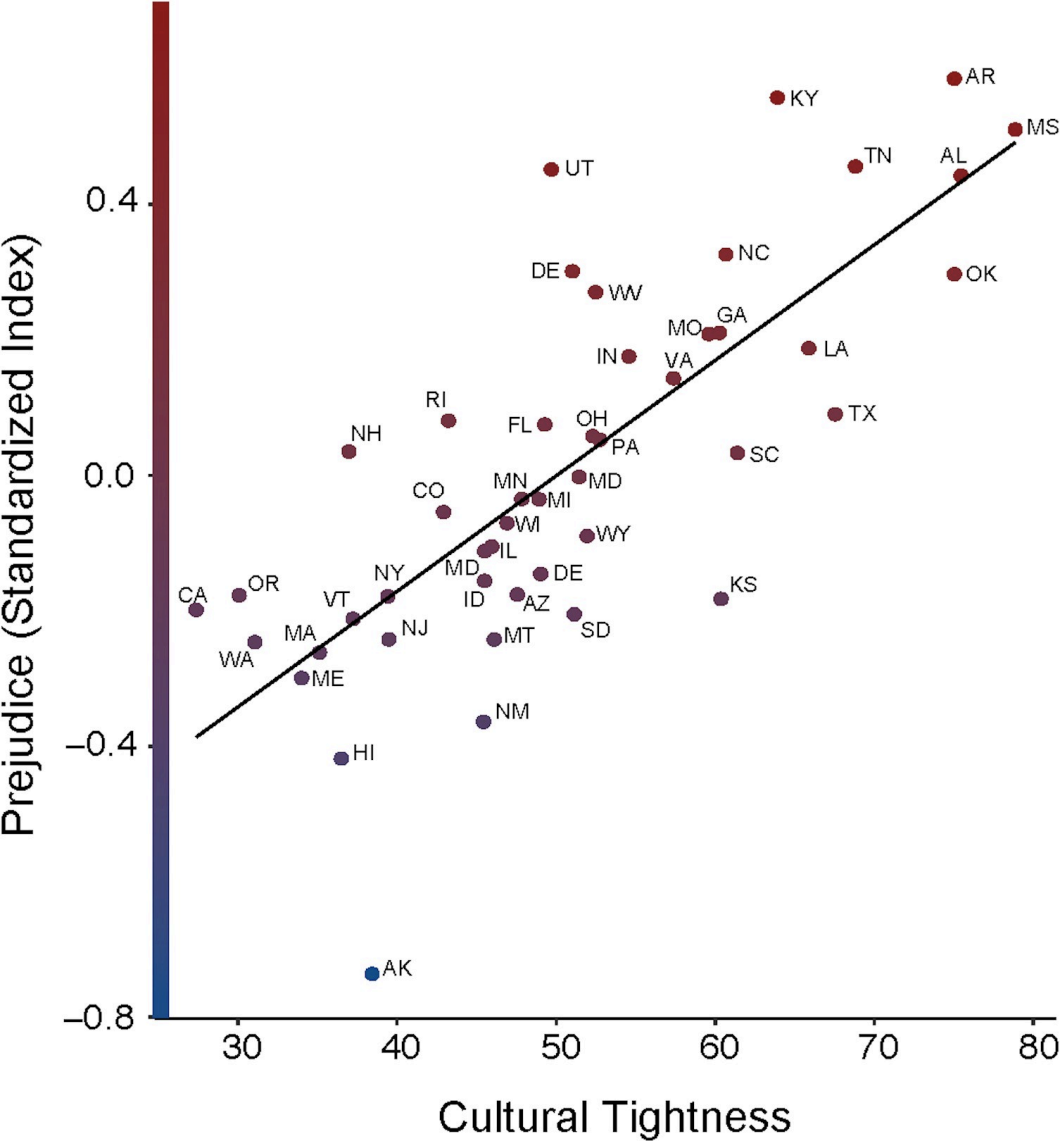
The relationship between cultural tightness and prejudice. Prejudice has been standardized in this figure. Prejudice is operationalized through the intolerance of a close relative marrying Black, Asian, Hispanic, and Jewish individuals.

As in Study 1, our first prediction was that tightness would correlate with prejudice. In support of this prediction, tightness at the state-level was robustly linked with prejudice, *b* = .02, *SE* = .003, *t* = 7.19, *p* < .001, *R*^*2*^_level 2_ = .64, even controlling for income, conservatism, and residential mobility (see Fig 2). In fact, whereas conservatism alone was a significant predictor of prejudice, *b* = .15, *SE* = .04, *t* = 4.11, *p* < .001, *R*^*2*^_level 2_ = .05, including tightness and conservatism in the same model nullified the effect of conservatism on prejudice, *b* = -.05, *SE* = .03, *t* = -1.30, *p* = .20, *R*^*2*^_level 2_ = .02, suggesting that the link between political conservatism and explicit prejudice was significant by virtue of shared variance with cultural tightness. Cultural tightness significantly predicted each item in our prejudice scale, positively correlating with prejudice against African Americans, *b* = .02, *SE* = .003, *t* = 7.17, *p* < .001, *R*^*2*^_level 2_ = .63, Asians, *b* = .01, *SE* = .003, *t* = 3.94, *p* < .001, *R*^*2*^_level 2_ = .46, gay people, *b* = .02, *SE* = .004, *t* = 3.26, *p* = .002, *R*^*2*^_level 2_ = .39, Hispanic people, *b* = .01, *SE* = .003, *t* = 4.01, *p* < .001, *R*^*2*^_level 2_ = .40, and Jewish people, *b* = .01, *SE* = .003, *t* = 2.97, *p* = .005, *R*^*2*^_level 2_ = .36.

#### Ecological threat, cultural tightness, and prejudice

We next predicted that tightness would mediate the association between ecological threat and prejudice. Ecological threat was significantly associated with both cultural tightness, *b* = 4.14, *SE* = .05, *t* = 91.48, *p* < .001, *R*^*2*^ = .04, and prejudice, *b* = .12, *SE* = .03, *t* = 3.51, *p* = .001, *R*^*2*^_level 2_ = .29, across states. Moreover, when cultural tightness and ecological threat were modeled simultaneously, tightness significantly predicted prejudice, *b* = .02, *SE* = .003, *t* = 5.54, *p* < .001, *R*^*2*^_level 2_ = .50. Our data therefore met the conditions for probing mediation [47], and a Monte Carlo simulation confirmed that cultural tightness mediated the relationship between ecological threat and prejudice, 95% *CIs* [.02, .20]. Ecological threat no longer significantly predicted prejudice when tightness was added to the model, indicating full mediation, *b* = .005, *SE* = .003, *t* = 0.15, *p* = .88, *R*^*2*^_level 2_ = .03.

## Study 3: Implicit prejudice across American states

Study 3 extended the findings of Study 2 to *implicit* prejudice, which we operationalized through bias in an implicit association test. This analysis allowed us to test whether cultural tightness was associated with automatic biases tapping cognitive associations between minority groups and negativity, adding to a growing interest in the factors explaining variance in implicit bias across cultural groups [51–52]. We tested for the relationship between cultural tightness and implicit bias using publicly available data from Project Implicit, a web-based platform where people can freely take a variety of implicit association tests (IATs) [53], with their time- and location-stamped data stored for analysis.

We included data from 3,855,737 US residents between 2002 and 2016 who had taken IATs related to homophobia (*n* = 1,003,773), and African American vs. Caucasian bias (*n* = 1,040,562)—mirroring two of our Study 2 analyses. We analyzed tests measuring participants’ implicit association between “bad” and (a) gay people, and (b) African Americans using the indices provided by Project Implicit. Other IATs exist that assess anti-elderly bias, anti-fat bias, and anti-disability bias. We excluded these from our central analyses because they seemed less characteristic of the prejudice we documented in Studies 1–2—and they are not included in Goffman’s tribal stigmas [41]. Nevertheless, we report the association between cultural tightness and these tests in the supplemental materials.

Analyses controlled for income and political conservatism using the same measures as in Study 2. As in Studies 1 and 2, our primary tests concerned a composite prejudice index that averaged across specific cases of prejudice, but we also report the link between statewide tightness and each specific form of prejudice. Unlike Studies 1 and 2, our two prejudice measures came from two independent sources of data, and could only be modeled together at the state level. We therefore performed general linear models rather than multi-level models.

### Results

#### Cultural tightness and prejudice

**Fig 3:**
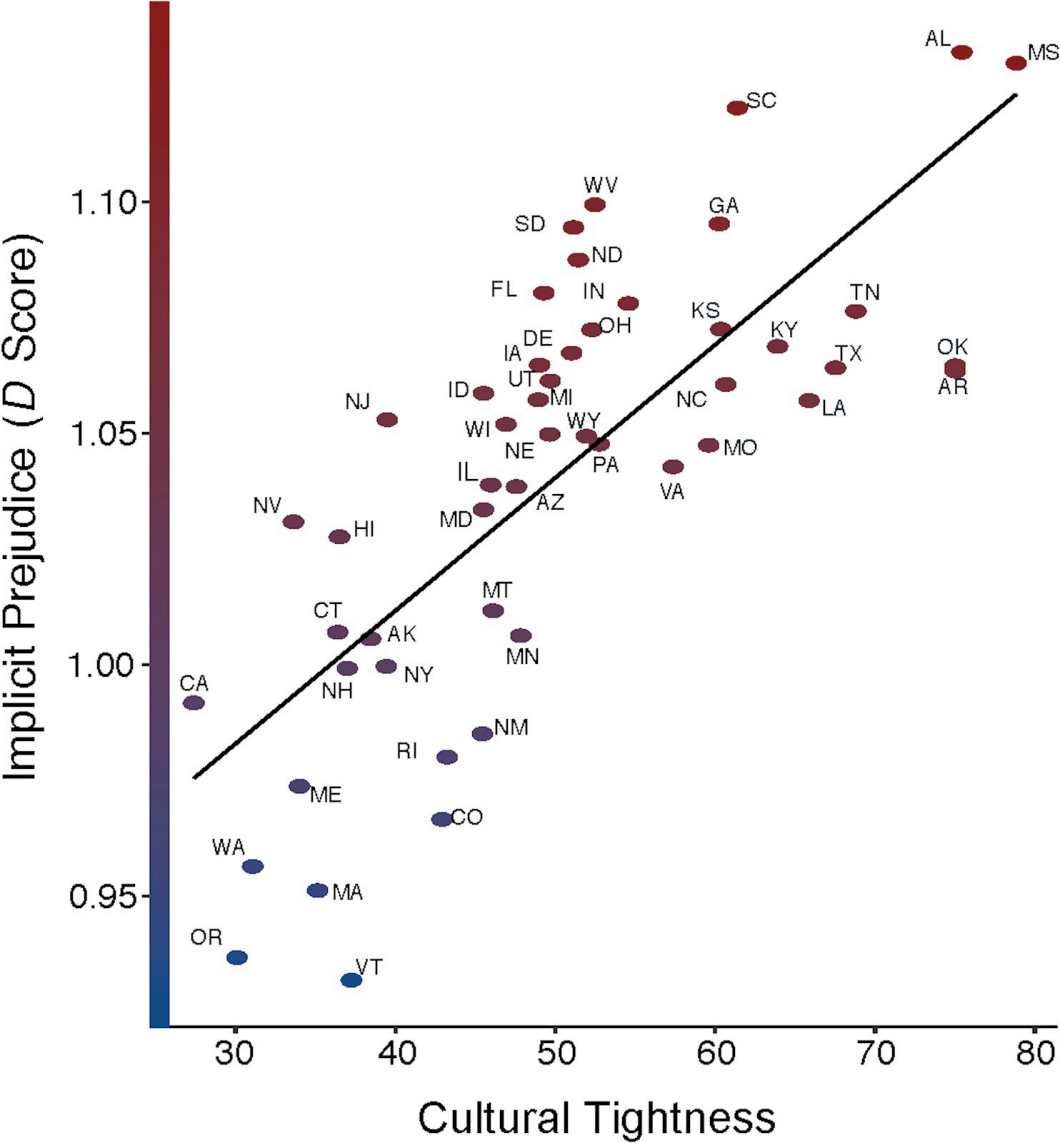
The relationship between cultural tightness and implicit prejudice. Implicit prejudice has been standardized in this figure.

As in Studies 1 and 2, our first prediction—that cultural tightness would be positively associated with implicit prejudice—was supported, *b* = .002, *SE* = .001, *t* = 3.82, *p* < .001, *R*^*2*^ = .11 (see Fig 3). This relationship also reached significance when our overall implicit prejudice index was decomposed into implicit racism, *b* = .005, *SE* = .001, *t* = 4.48, *p* < .001, *R*^*2*^_level 2_ = .32, and implicit anti-gay prejudice, *b* = .02, *SE* = .006, *t* = 3.04, *p* = .004, *R*^*2*^_level 2_ = .15. Unlike Study 2’s effects on explicit prejudice, conservatism also significantly and positively predicted implicit prejudice when modeled alongside cultural tightness, *b* = .02, *SE* = .007, *t* = 2.85, *p* = .007, *R*^*2*^ = .06.

#### Ecological threat, cultural tightness, and prejudice

Our second prediction—that cultural tightness would mediate the relationship between ecological threat and prejudice—was also supported. Ecological threat was associated with both cultural tightness, *b* = 7.64, *SE* = 1.45, *t* = 5.26, *p* < .001, *R*^*2*^ = .18, and implicit prejudice, *b* = .02, *SE* = .007, *t* = 2.47, *p* = .02, *R*^*2*^ = .05, but the link between tightness and prejudice remained significant when controlling for threat, *b* = .002, *SE* = .001, *t* = 2.74, *p* = .009, *R*^*2*^ = .06. A Monte Carlo simulation confirmed significant mediation, and ecological threat did not reach significance in this model, indicating full mediation, CIs [.01,.04], *b* = .03, *SE* = .08, *t* = 0.39, *p* = .70, *R*^*2*^ < .01.

## Study 4: Prejudice across historical societies

Study 4 tested for whether the link between cultural tightness and prejudice replicated in non-industrial societies, rather than just in current-day nations and states in the USA. We sampled 47 societies from the Standard Cross-Cultural Sample, which was built to be as geographically diverse as possible in order to minimize the probability of cross-cultural correlations being due to shared cultural ancestry or intercultural borrowing. Using a common technique in anthropology [54], we trained research assistants to read through ethnographies and assign societies scores ranging from 1–4 to indicate cultural tightness in six domains of life: socialization, gender, sexuality, marriage, institutional control, and funerals and mourning (our coding manual and coding notes are hosted on https://osf.io/2gk4v/). Codes across each of these domains converged towards a 1-factor solution and showed high reliability (Cronbach’s α = .87), and thus we averaged them into a single index representing the overall cultural tightness in each society.

**Fig 4:**
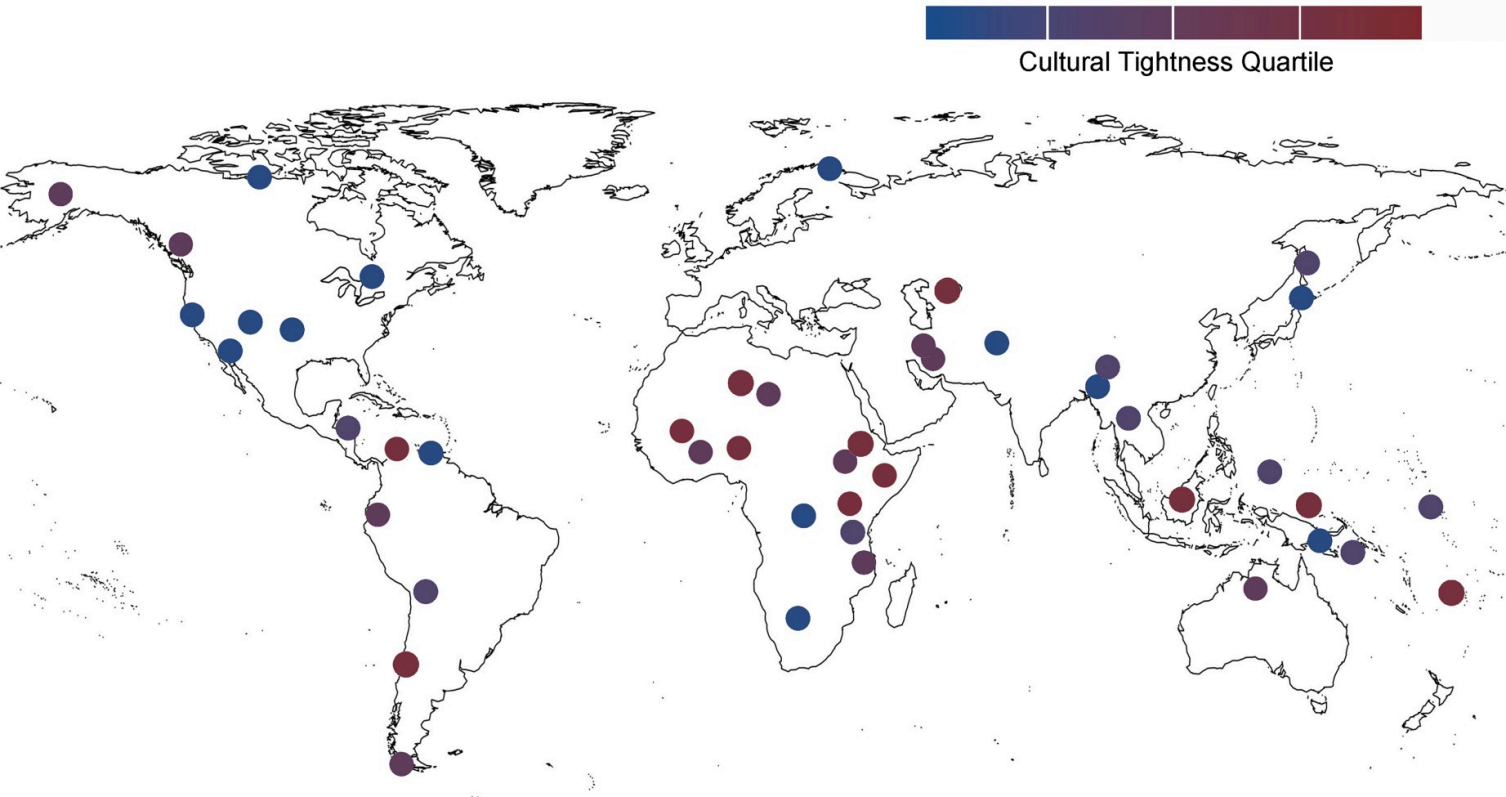
The sample of societies in Study 4. Each node represents a society. Societies are shaded based on their cultural tightness quartile such that the loosest societies are blue and the tightest societies are red.

We measured prejudice using Ross’s [55] cross-cultural codes assessing hostility towards other societies and acceptability of violence towards other societies. These variables correlated highly (*r* = .74) and so we averaged them into a composite prejudice index. Each society’s level of tightness and prejudice is displayed in Fig 4, and reported in the Supplementary Materials. We measured ecological threat by standardizing and summing codes that past studies developed to measure the severity of famine [56], pathogen prevalence [57], overpopulation [58], and warfare [55]—the same threats included in the indices from Studies 1–3.

Sampling societies from the ethnographic record also gave us the opportunity to control for shared cultural ancestry in our analyses by randomly varying intercepts across each society’s language family membership in multi-level models. As in Studies 2–3, Study 4 also controlled for residential mobility. We derived language family membership from D-PLACE [59].

### Results

#### Cultural tightness and prejudice

We first tested our prediction that cultural tightness would correlate with prejudice across non-industrial societies. This prediction was supported, *b* = .85, *SE* = .28, *t* = 2.98, *p* = .005, *R*^*2*^_level 1_ = .09. Even controlling for shared heritage, tightness was associated with greater prejudice, replicating our pattern of results in current-day groups.

#### Ecological threat, cultural tightness, and prejudice

We next tested our prediction that cultural tightness would mediate the relationship between ecological threat and prejudice. Consistent with Studies 2–3, ecological threat predicted both cultural tightness, *b* = .25, *SE* = .13, *t* = 1.92, *p* = .05, *R*^*2*^_level 1_ = .03, and prejudice, *b* = 1.25, *SE* = .20, *t* = 6.18, *p* < .001, *R*^*2*^_level 1_ = .36. Tightness remained a significant predictor of prejudice when it was modeled alongside threat, *b* = .50, *SE* = .23, *t* = 2.19, *p* = .03, *R*^*2*^_level 1_ = .08, and a Monte Carlo simulation confirmed significant mediation, 95% *CIs* [.02, .37]. Threat also remained a significant predictor in this model, *b* = 1.12, *SE* = .20, *t* = 5.56, *p* < .001, *R*^*2*^_level 1_ = .35, indicating partial mediation.

## Study 5: Experimentally evoked support for cultural tightness and prejudice

Study 5 built on the correlational evidence in Studies 1–4 with an experiment that manipulated the salience of ecological threat and measured how this changed people’s support for cultural tightness and prejudice. We note that support for cultural tightness is not the same as living in a tight society. Indeed, a person may live in a loose society and support a tighter society, or vice versa. However, since tightness is a group-level metric, support for cultural tightness provides an approximation that we predict should be associated at the individual level with many correlates of cultural tightness at the group level.

This study also allowed us to test whether manipulating *perceived* threat has the same effects on cultural tightness and prejudice as the actual threats that we measured in Studies 1–3. We hypothesized that making ecological threats facing society salient would increase individuals’ support for cultural tightness, and that this would in turn increase prejudice.

Study 5 sampled 1049 people (617 women, 432 men; *M*_age_ = 26.03, *SD*_age_ = 14.89; *M*_*SES*_ = 6.11 on an 11-point scale, *SD*_*SES*_ = 2.17; 238 Catholics, 212 Protestants, 93 Buddhists, 11 Hindus, 7 Jews, 19 Muslims, 45 Agnostic, 228 “none”, 124 “other,” 70 Atheists, 2 religion not reported) from four nations (Singapore, Germany, USA, Brazil) that we recruited through Qualtrics panels. We chose two loose nations (USA, Brazil) and two tight nations (Germany, Singapore) from Gelfand and colleagues’ [24] survey in order to test whether perceived threat would show the same effects in tight and loose cultures. We had no a priori hypotheses about whether the effect would vary across tight and loose cultures, but we chose to test for this interaction for the sake of comprehensiveness.

The study had three conditions. In the ecological threat condition, participants were prompted to describe their most salient ecological threat and were given “a foreign attack” and “major recession” as examples. In the control condition, participants were prompted to describe what they had for breakfast. We also included a negativity control condition where participants described a negative event in their personal life, such as “failing to achieve goals,” and “failing to keep up with workload.”

All participants were then prompted to endorse an ending to 10 incomplete statements concerning their support for cultural tightness. For example, in one item, participants completed the sentence “My country is currently…” with a response on a 1–9 scale anchored at “not permissive enough” (1) and “too permissive” (9). Each of these statements is included in Table 1. Finally, participants indicated their agreement with 5 statements measuring prejudice (e.g. “when jobs are scarce, employers should give priority to people from my country over immigrants”) used in previous major surveys and cultural psychology studies on prejudice [60]. Participants rated their agreement with these items using a 1–7 scale anchored at “Strongly Disagree” and “Strongly Agree.”

**Table 1:** Items in support for cultural tightness scale.

Item	Low Anchor	High Anchor
My country is currently…	Not Permissive Enough	Too Permissive
People in my country are currently…	Overly adherent of my country’s customs	Overly ignorant of my country’s customs
People in my country…	Follow the rules too much	Don’t follow the rules enough
My country currently has…	Too many rules	Too few rules
Social norms in my country are…	Too rigid	Too flexible
People in my country who break the law are currently…	Punished too often	Punished too rarely
Criminal punishment in my country is currently…	Too harsh	Too lenient
My country’s norms are currently	Enforced too strictly	Not enforced strictly enough
People who don’t conform to the norms in my country are…	Treated too harshly	Treated too kindly
My country is currently…	Too tight	Too loose

**Ethical approval and informed consent.** Studies 5–7 involved human subjects, and were approved by the University of Maryland Institutional Review Board. All subjects completed informed consent forms.

### Results

#### Experimentally induced threat and support for cultural tightness

Did our manipulation of threat affect people’s support for cultural tightness? An ANOVA identified a significant omnibus effect, *F*(2,1046) = 5.18, *p* = .006, such that participants exposed to ecological threat held more favorable attitudes towards tightness than participants in the neutral control, *M*_diff_ = .36, Tukey HSD *p* = .01, or in the negativity control, *M*_diff_ = .32, Tukey HSD *p* = .02. A follow-up multiple regression with dummy-coded condition variables found that the effect of ecological threat vs. control did not vary significantly across tight and loose nations, *b* = .19, *SE* = .23, *t* = 0.83, *p* = .41, *R*^*2*^ < .001, and neither did the effect of ecological threat vs. negativity control, *b* = -.36, *SE* = .23, *t* = -1.60, *p* = .11, *R*^*2*^ = .001.

#### Cultural tightness and prejudice

We next tested whether people’s support for cultural tightness predicted their prejudice. In a general linear model, support for cultural tightness did indeed predict higher prejudice, *b* = .06, *SE* = .02, *t* = 2.37, *p* = .01, *R*^*2*^ = .01. This effect did not significantly vary across tight and loose countries, *b* = -.09, *SE* = .05, *t* = -1.70, *p* = .09, *R*^*2*^ = .002. However, nation-level cultural tightness did predict people’s prejudice, *b* = .66, *SE* = .09, *t* = 7.59, *p* < .001, *R*^*2*^ = .05, such that people in tighter nations were more prejudiced than people in looser nations.

#### Ecological threat, cultural tightness, and prejudice

Finally, we tested our second prediction that support for cultural tightness would mediate the indirect effect of threat on prejudice. In this analysis, we collapsed across the two control conditions (which showed similar effects in our previous analysis) and contrasted them with the ecological threat condition. As predicted, support for cultural tightness was positively associated with prejudice, *b* = .13, *SE* = .03, *t* = 4.73, *p* < .001, *R*^*2*^ = .02, while controlling for experimental condition and nation-level cultural tightness. A Monte Carlo simulation supported a significant indirect effect, 95% *CIs* [.02, .14].

Interestingly, ecological threat had no total effect on prejudice, *b* = .06, *SE* = .09, *t* = .68, *p* = .50, *R*^*2*^ < .001, which suggests that priming threat alone was not enough to increase prejudice; rather, priming threat needed to accompany an increase in support for cultural tightness in order to increase prejudice. Although measuring a mediator cannot establish a causal relationship on an outcome variable, this analysis strongly suggests that threat salience only affects prejudice to the extent that it affects the support for cultural tightness.

## Studies 6–7: Support for tightness, prejudice, and voting intentions

Our final two studies adopted a broader perspective, focusing not only on people’s prejudiced attitudes towards minorities, but also their voting intentions for nationalist political candidates. Politics play an important role in cultural change, and many historical cases of intergroup hostility have been rooted in prejudiced political leaders and governments [61]. We theorized that individuals who feared ecological threat and desired tighter cultural norms would be especially likely to support these kinds of political figures.

Data from both studies came from real election cycles: the 2016 election cycle within the United States and the 2017 election cycle within France. We chose these election cycles because they featured candidates who advocated policies that put restrictions on minority groups. In the 2016 American election, Donald Trump recommended a border wall with Mexico, a travel prohibition on individuals from predominantly Muslim nations, and surveillance of Mosques within the United States. In the 2017 French election, Marine Le Pen recommended a moratorium on many forms of legal immigration and the closing of Mosques around France.

Study 6 surveyed 562 Americans (289 women, 273 men; *M*_age_ = 29.85, *SD*_age_ = 16.82) during the United States 2016 primary election using a panel method that ensured our sample was nationally representative in terms of race, political affiliation, and region. Study 7 surveyed 320 French respondents (118 women, 202 men; *M*_age_ = 23.81, *SD*_age_ = 14.93) on April 20^th^, 2017—three days before the first round of the election. Participants rated their concern about 10 different ecological threats (e.g. natural disaster, debt, an attack from North Korea). Ratings of ecological threats were reliable amongst both American (Cronbach’s α = .89) and French (Cronbach’s α = .84) participants, so we averaged them into a “perceived ecological threat” composite score. We also gauged participants’ support for cultural tightness and their prejudice (using the same measures from our previous study), and asked participants who they planned to vote for in the upcoming election. Study 6’s analyses of American participants modeled intercepts as randomly varying across participants’ states, given state-level differences in tightness from Study 2. Study 6 also controlled for participants’ prior political orientation using a 1 (“Democrat”) to 5 (“Republican”) scale.

### Results

#### Cultural tightness and prejudice

Consistent with our first prediction, support for cultural tightness predicted prejudice among both American, *b* = .26, *SE* = .04, *t* = 7.05, *p* < .001, and French, *b* = .37, *SE* = .05, *t* = 7.83, *p* < .001, participants.

#### Ecological threat, cultural tightness, and prejudice

Perceived threat was associated with cultural tightness for both American, *b* = .17, *SE* = .07, *t* = 2.21, *p* = .02, *R*^*2*^_level 1_ = .01, and French, *b* = .40, *SE* = .09, *t* = 4.04, *p* < .001, *R*^*2*^ = .05, participants. Perceived threat also predicted prejudice for both American, *b* = .66, *SE* = .06, *t* = 10.64, *p* < .001, *R*^*2*^_level 1_ = .17, and French, *b* = .66, *SE* = .09, *t* = 7.62, *p* < .001, *R*^*2*^_level 1_ = .15, participants. As predicted, support for cultural tightness mediated the association between perceived threat and prejudice amongst Americans, 95% *CIs* [.02, .08], and French participants, 95% *CIs* [.12, .42]. The relationship between perceived threat and prejudice remained significant controlling for support for cultural tightness amongst American, *b* = .17, *SE* = .07, *t* = 2.21, *p* = .02, *R*^*2*^_level 1_ = .01, and French, *b* = .53, *SE* = .08, *t* = 6.45, *p* < .001, *R*^*2*^_level 1_ = .10, participants, indicating partial mediation.

#### Voting preferences

We next analyzed voting preferences. Amongst Americans who indicated an intention to vote for a candidate (*N* = 419), 29% planned to vote for Donald Trump, 5% for John Kasich, 12% for Ted Cruz, 27% for Hilary Clinton, and 25% for Bernie Sanders. Intention to vote for Donald Trump was associated with perceived threat, *OR* = 1.39, *Z* = 2.68, *p* = .007, cultural tightness, *OR* = 1.40, *Z* = 4.83, *p* < .001, and prejudice, *OR* = 1.86, *Z* = 6.57, *p* < .001. Among French participants who indicate an intention to vote for a candidate (*N* = 273), 29% planned for vote for Marine LePen, 28% planned to vote for Melanchon, 25% planned to vote for Macron, 11% planned to vote for Fillon, and 8% planned to vote for Hamon. As with Donald Trump, intention to vote for Marine LePen was predicted by perceived threat, *OR* = 1.47, *Z* = 2.37, *p* = .02, support for cultural tightness, *OR* = 1.91, *Z* = 5.95, *p* < .001, and prejudice, *OR* = 2.74, *Z* = 6.96, *p* < .001.

**Fig 5:**
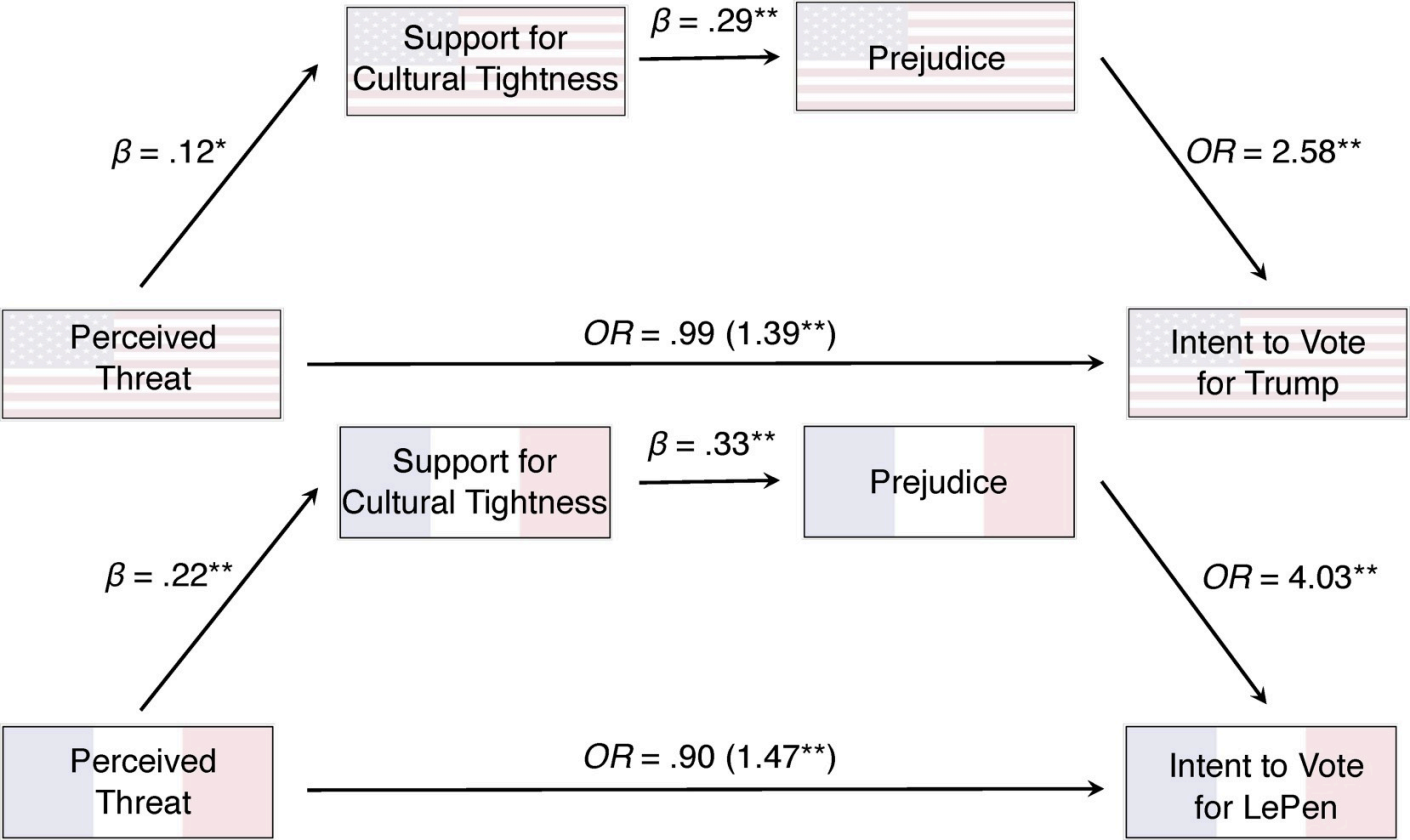
A serial mediation path model showing the effects of threat on intention to vote for Donald Trump (above) and Marine LePen (below) via support for cultural tightness and prejudice. All effects have been standardized so they can be interpreted as effect sizes. Single-starred associations are significant at the *p* < .05 level; double-starred associations are significant at the *p* < .005 level. The X-Y path inside the parentheses is the total effect, whereas the effect outside parentheses is the direct effect.

We fit two serial mediation path models estimating the effect of threat on support for Donald Trump (among Americans) and Marine LePen (among French) via individuals’ support for cultural tightness and their prejudiced attitudes. Both of these path models—displayed in Fig 5—showed significant indirect effects (American 95% CIs [.001, .008]; French 95% CIs [.006, .02]), but no significant direct effects (American 95% CIs [-.05, .04]; French 95% CIs [-.09, .02]), suggesting that threat did not predict political preference after controlling for tightness and prejudice. The reverse mediational pathways had no significant indirect effect. These findings show that voters who believed their country was under threat were more likely to vote for Donald Trump and Marine LePen, and their preferences could be fully explained by their support for cultural tightness and prejudice. Our supplemental materials summarize how threat and support for tightness related to support for other candidates.

## Discussion

Seven studies explored the ecological and cultural foundations of prejudice. We found that ecological threats such as pathogens, warfare, and resource scarcity predicted greater cultural tightness, and people in tighter cultures were more prejudiced against racial, national, sexual, and religious minorities. These relationships replicated across current-day nations (Study 1), US federal states (Studies 2–3), and non-industrial societies (Study 4), manifested through both explicit (Studies 1–2, 4) and implicit (Study 3) prejudice, and replicated even when controlling for other structural and attitudinal factors such as economic development, inequality, residential mobility, conservatism, and shared cultural heritage.

Our final three studies showed that support for perceptions of societal threats influenced people’s support for cultural tightness, in turn predicting prejudiced attitudes and xenophobic political preferences. In Study 5, experimentally increasing the salience of societal threats increased support for strong norms, which correlated with greater prejudice. Studies 6–7 used a correlational design to replicate these effects and tie them to support for two politicians—Donald Trump and Marine LePen—who advanced prejudicial policy positions in recent elections. Studies 6–7 replicated controlling for self-identified political conservatism.

While our primary results involved prejudice against people with different social identities, our supplementary materials present further evidence for a relationship between tightness and other forms of prejudice, including a 20-nation field study that documents prejudice against individuals with physical stigmas.

Our results do not mean that cultural tightness is the only cultural factor linked with prejudice. Factors such as religion, inequality, and resource scarcity may predict prejudice and discrimination across cultures [23,62–64]. Our correlational studies also do not show that tightness causes prejudice. However, the link between prejudice and cultural tightness in our studies replicated controlling for these factors, suggesting that the connection between tightness and prejudice cannot be reduced to other sociocultural factors.

We encourage future research to explore this link with other emerging cross-cultural methods, such as time series and phylogenetic analysis [62,65]. We also encourage future studies to test for potential dynamical feedback between cultural tightness and prejudice: Prejudice may feed back into tightness over time as politicians use intergroup threats to advance culturally tight policy positions. We did not see evidence of this mutual influence in our analysis, but it may be detectable with more intensive longitudinal designs which can directly probe for the causality between ecological threats tightness, and prejudice. Finally, we encourage research to test whether tightness is linked with group polarization as well as prejudice, since political polarization has risen in recent years in the United States [66].

More generally, our findings speak to a recent rise in policies and governments with agendas that are hostile to ethnic minorities and immigrants. Although in many cases explicit and implicit bias against minorities are decreasing [67], there is nevertheless a trend toward nationalist governments and policies in recent years. This trend has potentially resulted in support for programs such as Brexit in England, a border wall in the United States, and far-right nationalist parties in Austria, Hungary, and Poland, among other European nations. Our studies show that these events are neither isolated nor random, but instantiations of a broader principle that echoes across human history: Perceptions of ecological threat tighten societies, which in turn is linked to order and social coordination, but also to hostility and intolerance.

## Supporting information

S1 File Supplemental materials, including supplemental tables (A-F) and supplemental figures (A-B).
(DOCX)
