# Supplementary material for: Ecological and cultural factors underlying the global distribution of prejudice
Source: PLoS One. 2019 Sep 6;14(9):e0221953. doi: 10.1371/journal.pone.0221953 (PMC6730889; doi:10.1371/journal.pone.0221953)
Supplement: S1 File — Supplemental materials, including supplemental tables (A-F) and supplemental figures (A-B). (DOCX) [file pone.0221953.s001.docx]

**Supporting Information**

**Overview of Supporting Information**

These Supporting Information contain data and analyses that extend our primary findings, but that space limitations did not permit in our main text. This includes (a) analyses of additional items within the World Values Survey and the General Social Survey (Studies 1-2), (b) analyses of additional implicit association test data (Study 3), a full listing of historical societies by strength of norms and prejudice (Study 4), analyses of how perceived threat, support for strong norms, and prejudice predicted all candidates in the 2016 American election and the 2017 French election (Studies 6-7), and a supplemental field experiment examining whether the strength of cultural norms predicts discrimination on the basis of physical stigmas across current-day nations.

**Study 1 Supplemental Information**

Study 1 sought to identify whether the strength of norms was associated with various forms of prejudice across nations, and whether strength of norms mediated a significant indirect effect between nations’ historical levels of threat and their self-reported prejudice. Our primary items in this analysis were 7 items that asked respondents if they would refuse to live next to individuals from a variety of out-groups. However, we also analyzed several other items that appeared face valid measures of prejudice, and were asked in at least 10 nations in order to appropriately fit multi-level models. We present the results from these analyses in Supplemental Table 1. Multi-level models were fit with the same parameters as the multi-level models that we describe in Study 1’s main text.

| **Supplemental Table 1. Associations Between the Strength of Norms, Perceived Threat, and Forms of Prejudice Across Current-day Nations** | | | | | |
| --- | --- | --- | --- | --- | --- |
| **Variable** | **Coding Scheme** | **Threat** | | **Strength of Norms** | |
|  |  | *b (SE)* | *R^2^* | *b (SE)* | *R^2^* |
| Trust: People of a different nationality | 1 (Trust Completely) – 5 (Not Trust at All) | .02 (.01)† | .07 | 05 (.02)**^a^ | .20 |
| Trust: People of a different religion | 1 (Trust Completely) – 5 (Not Trust at All) | .02 (.01) | .11 | .04 (.02)* | .12 |
| Immigration policy | 1 (Let everyone come) – 4 (Strict Prohibitions) | -.002 (.01) | < .01 | .03 (.01)†^a^ | .11 |
| Justifiable: Sex before marriage | 1 (Never Justifiable) – 10 (Always Justifiable) | -.09 (.06) | .19 | -.41 (.08)**^a^ | .57 |
| Justifiable: Divorce | 1 (Never Justifiable) – 10 (Always Justifiable) | .03 (.01)** | .42 | .05 (.02)*^a^ | .19 |
| Justifiable: Prostitution | 1 (Never Justifiable) – 10 (Always Justifiable) | -.03 (.02) | .12 | -.14 (.05)* | .21 |
| Justifiable: Homosexuality | 1 (Never Justifiable) – 10 (Always Justifiable) | -.03 (.03) | .32 | -.21 (.08)* | .18 |
| The only acceptable religion is my religion | 1 (Strongly Agree) – 4 (Strongly Disagree) | .03 (.01)** | .42 | .05 (.02)*^a^ | .19 |

*Note. R^2^* values correspond to level-2 variance explained.

† indicates an effect that is between *p* = .05 and *p* = .10

*indicates significance at *p* = .05 level.

** indicates significance at *p* =.005 level.

*^a^*Strength of norms fully mediates an indirect effect of threat on prejudice

**Study 2 Supplemental Information**

Study 1 sought to identify whether the strength of norms was associated with various forms of prejudice across nations, and whether strength of norms mediated a significant indirect effect between nations’ historical levels of threat and their self-reported prejudice. Our primary items in this analysis were 7 items that asked respondents if they would refuse to live next to individuals from a variety of out-groups. However, we also analyzed several other items that appeared face valid measures of prejudice, and were asked in at least 10 nations in order to appropriately fit multi-level models. We present the results from these analyses in Supplemental Table 2. Multi-level models were fit with the same parameters as the multi-level models that we describe in Study 1’s main text.

| **Supplemental Table 2. Associations Between the Strength of Norms, Perceived Threat, and Forms of Prejudice Across USA States** | | | | | |
| --- | --- | --- | --- | --- | --- |
| **Variable** | **Coding Scheme** | **Threat** | | **Strength of Norms** | |
|  |  | *b (SE)* | *R^2^* | *b (SE)* | *R^2^* |
| Improving the conditions of Blacks | 1 (Too Little) – 2 (Too Much) | .12 (.01)** | .70 | .01 (.001)***^c^* | .68 |
| Allow homosexual to speak | 1 (Allowed) – 2 (Not Allowed) | .11 (.01)** | .48 | .01 (.001)***^d^* | .67 |
| Allow homosexual books in library | 1 (Remove) – 2 (Do Not Remove) | -.05 (.02)** | .19 | -.01 (.001)***^d^* | .66 |
| Favor law against racial intermarriage | 1 (Yes) – 2 (No) | -.08 (.02)** | .24 | -.01 (.001)***^d^* | .51 |
| Blacks shouldn’t push for recognition | 1 (Agree Strongly) – 4 (Disagree Strongly) | -.17 (.05)** | .21 | -02 (.003)***^d^* | .38 |
| Whites have a right to segregated neighborhoods | 1 (Agree Strongly) – 4 (Disagree Strongly) | -.20 (.05)** | .34 | -.02 (.002)***^d^* | .52 |
| Should Whites and Blacks go to the same school? | 1 (Same Schools) – 2 (Separate School) | .08 (.01)** | .54 | .005 (.001)***^c^* | .52 |
| How close do respondents feel to Black people | 1 (Not at all close) – 2 (Very close) | .17 (.05)** | .25 | .003 (.004) | < .01 |
| Homosexual sex relations | 1 (Always Wrong) – 4 (Not Wrong at All) | -.17 (.06)** | .30 | -.02 (.003)***^d^* | .74 |
| Increase or decrease immigration to the USA | 1 (Should be Increased a Lot) – 5 (Should be Increased a Lot) | .07 (.03)* | .22 | .005 (.001)***^d^* | .92 |
| Are immigrants demanding too many rights? | 1 (Strongly Agree) – 5 (Strongly Disagree) | -.10 (.05)† | .15 | -.01 (.003)† | .14 |
| Government attention for Black people | 1 (Much More) – 5 (Much Less) | -.22 (.06)** | .52 | -.01 (.004)** | .25 |
| Living in a neighborhood where half your neighbors were _____*^a^* | 1 (Strongly Favor) – 5 (Strongly Oppose) | .18 (.04)** | .39 | .02 (.002)** | .77 |
| Influence of _______*^b^* | 1 (Too Much Influence) – 4 (Not Enough Influence) | -.13 (.03)** | .72 | -.008 (.001)** | .50 |

*Note. R^2^* values correspond to level-2 variance explained.

† indicates an effect that is between *p* = .05 and *p* = .10

*indicates significance at *p* = .05 level.

** indicates significance at *p* =.005 level.

*^a^*This was a composite score derived from questions where participants rated how they would feel about living in a neighborhood with more than half Blacks, Asians, Jews, and Hispanics neighbors (α = .83).

*^b^*This was a composite score derived from questions where participants rated whether Blacks, Asians, Jews, and Hispanics had too little influence or too much influence in the country (α = .65).

*^e^*Partially mediates an indirect effect of threat on prejudice

*^d^*Fully mediates an indirect effect of threat on prejudice

**Study 3 Supplemental Information**

Study 3 tested whether strength of norms and historical threat across the 50 American states could predict implicit prejudice. We operationalized prejudice in Study 4 through scores on the White vs. Black implicit association test (IAT) and the heterosexual vs. homosexual IAT, which were freely available on Project Implicit. Project implicit also had IATs available measuring anti-elderly bias and anti-disability bias^[[1]](#footnote-1)^. These tests did not conform to our operationalization of prejudice through Goffman’s tribal identity list, but they did measure bias against commonly stigmatized groups in America, so we included them in a supplemental analysis using the same analytic procedure as in our main analyses to test their association with strength of states’ norms (including control variables). The results, which we summarize in Supplemental Table 3, revealed that strength of norms and threat was associated with both implicit anti-elderly bias and anti-disability bias.

| **Supplemental Table 3. Associations Between the Strength of Norms and Implicit Prejudice** | | | | |
| --- | --- | --- | --- | --- |
| **Variable** | **Threat** | | **Strength of Norms** | |
|  | *b (SE)* | *R^2^* | *b (SE)* | *R^2^* |
| Elderly people | .02 (.004)** | .30 | .01 (.003)** | .13 |
| Disabled people | .01 (.004)* | .07 | .01 (.005)** | .19 |

*Note. R^2^* values correspond to level-2 variance explained.

*indicates significance at *p* = .05 level.

** indicates significance at *p* =.005 level.

**Study 4 Supplemental Information**

Study 4 tested whether historical societies with stronger norms also had greater rates of prejudice. Supplemental Table 4 list all societies (*n* = 45) in this analysis by their strength of norms and prejudice. All variables in Supplemental Table 4 have been standardized.

| **Supplemental Table 4. Strength of Norms and Prejudice by Society** | | |
| --- | --- | --- |
| **Society Name** | **Prejudice** | **Strength of Norms** |
| Copper Eskimo | -1.59 | -2.78 |
| Mbuti | -1.59 | -2.47 |
| Lapps | -1.59 | -1.48 |
| Omaha | 0.53 | -1.44 |
| Warrau | -0.74 | -1.44 |
| Burusho | 0.11 | -1.36 |
| Havasupai | -0.32 | -1.11 |
| Ainu | 0.53 | -0.92 |
| Orokaiva | 0.96 | -0.85 |
| Kung | -1.59 | -0.79 |
| Lakher | 0.96 | -0.79 |
| Klamath | 0.53 | -0.70 |
| Pawnee | 0.53 | -0.70 |
| Papago | -0.74 | -0.70 |
| Trobrianders | -1.59 | -0.41 |
| Tikopia | -1.59 | -0.41 |
| Rwala Bedouin | 0.53 | -0.37 |
| Miskito | 0.96 | -0.26 |
| Yapese | 0.11 | -0.17 |
| Vietnamese | -1.59 | 0.01 |
| Khalka Mongols | -1.59 | 0.11 |
| Aymara | 0.96 | 0.11 |
| Gilyak | -1.59 | 0.19 |
| Masai | 0.96 | 0.26 |
| Teda | 0.96 | 0.33 |
| Otoro Nuba | 0.11 | 0.36 |
| Cayapa | -1.17 | 0.36 |
| Tiwi | 0.96 | 0.44 |
| Basseri | 0.96 | 0.45 |
| Yahgan | -1.59 | 0.45 |
| Eyak | 0.11 | 0.54 |
| Tallensi | -0.74 | 0.55 |
| Nyakusa | 0.11 | 0.58 |
| Kurd | 0.96 | 0.63 |
| Bellacoola | -0.32 | 0.66 |
| Amhara | 0.96 | 0.78 |
| Mapuche | 0.96 | 0.77 |
| Iban/Sea Dayak | 0.96 | 0.80 |
| Bambara | 0.96 | 0.85 |
| Somali | 0.96 | 0.85 |
| Kazak | 0.96 | 0.98 |
| Goajiro | 0.11 | 1.22 |
| Ganda | 0.96 | 1.25 |
| Manus | -0.32 | 1.29 |
| Riffians | 0.53 | 1.34 |

*Note.* All scores have been standardized

**Study 6 Supplemental Information**

Study 6 had two primary aims: First, we replicated our mediational model, in which perceived threat predicted strength of norms, which in turn predicted self-reported prejudice. Second, we tested whether perceived threat, strength of norms, and prejudice predicted people’s likelihood of voting for Donald Trump. We focused on Donald Trump in these voting analyses because he advanced a uniquely ethnocentric rhetoric and policy recommendations during his campaign (e.g. building a border wall, monitoring mosques). Nevertheless, in supplemental analyses, we tested for whether perceived threat, strength of norms, and prejudice predicted participants’ likelihood of voting for other candidates.

Supplemental Table 5 lists the effect size (odds ratio) and significance (*p* value) concerning how our three independent variables predicted support for all 5 Republican and Democratic candidates who were eligible in the primaries at the time of our Study. Each variable has been estimated independently (rather than simultaneously in a multiple regression). These models revealed that perceived threat and strength of norms only positively predicted intentions to vote for Donald Trump, whereas prejudice predict voting for Donald Trump and Ted Cruz. In addition, perceived threat, strength of norms, and prejudice each negatively predicted intentions to vote for Bernie Sanders. Supplemental Figure 1 shows the mean perceived threat, cultural tightness, and prejudice characterizing each politician’s voter base.

| **Supplemental Table 5. Voter Intentions for American Presidential Candidates by Perceived Threat, Strength of Norms, and Prejudice** | | | |
| --- | --- | --- | --- |
| **Candidate** | **Perceived Threat** | **Strength of Norms** | **Prejudice** |
|  | *OR (z)* | *OR (z)* | *OR (z)* |
| Donald Trump | 1.39 (2.68)* | 1.40 (4.83)** | 1.86 (6.57)** |
| Ted Cruz | 1.00 (-.01) | 1.16 (1.61)† | 1.32 (2.42)* |
| John Kasich | 1.57 (1.72)† | 1.15 (1.07) | 1.08 (.50) |
| Hilary Clinton | 1.07 (.61) | .97 (-.58) | 1.05 (.71) |
| Bernie Sanders | .72 (-2.65)** | .60 (-6.64)** | .64 (-5.91)** |

† indicates an effect that is between *p* = .05 and *p* = .10

* indicates significance at *p* = .05 level.

** indicates significance at *p* =.005 level.

*Supplemental Figure 1.* The mean perceived threat, support for cultural tightness, and prejudice of participants who intended to vote for Democratic candidates (Bernie Sanders and Hilary Clinton) and Republican candidates (Ted Cruz, John Kasich, and Donald Trump).

**Study 7 Supplemental Information**

Study 7 had the same aims as Study 6, but we specifically examined support for Marine LePen, since she advanced more ethnocentric policy recommendations than the other candidates in the 2017 French election. Supplemental Table 6 summarizes how perceived threat, strength of norms, and prejudice predicted participants’ likelihood of voting for other candidates in this election. We used the same models as our Supplemental Study 6 analyses for these tests. Results revealed that perceived threat and prejudice positively predicted intentions to vote for Marine LePen and Francois Fillon, but support for strong norms uniquely predicted intentions to vote for LePen. Strength of norms also negatively predicted intentions to vote for Jean-Luc Melancon and Benoit Hamon, and Prejudice negatively predicted intentions to vote for Melanchon, Hamon, and Macron. Supplemental Figure 2 shows the mean perceived threat, cultural tightness, and prejudice characterizing each politician’s voter base.

| **Supplemental Table 6. Voter Intentions for French Presidential Candidates by Perceived Threat, Strength of Norms, and Prejudice** | | | |
| --- | --- | --- | --- |
| Candidate | **Perceived Threat**  *OR (z)* | **Strength of Norms**  *OR (z)* | **Prejudice**  *OR (z)* |
| Marine LePen | 1.47 (2.37)* | 1.95 (6.22)** | 2.74 (6.96)** |
| Francois Fillon | 1.76 (2.24)* | 1.30 (1.95)† | 1.81 (3.41)** |
| Emmanuel Macron | .87 (-.83) | .95 (-.56) | .80 (-2.26)* |
| Jean-Luc Melanchon | .75 (-1.79)† | .65 (-4.39)** | .70 (-3.62)** |
| Benoit Hamon | .87 (-.53) | .73 (-2.10)* | .72 (-2.20)* |

† indicates an effect that is between *p* = .05 and *p* = .10

* indicates significance at *p* = .05 level.

** indicates significance at *p* =.005 level.

**

*Supplemental Figure 2.* The mean perceived threat, support for cultural tightness, and prejudice of participants who intended to vote for (from top to bottom) Benoit Hamon, Jean-Luc Melanchon, Emmanuel Macron, Francois Fillon, and Marine LePen.

**Supplemental Study: Discrimination Against Physical Stigmas**

In addition to our primary research question concerning socioecological threat, prejudice, and prejudice, we also explored whether strength of norms can predict prejudice against individuals with physical stigmas. We include a brief description of this study’s aims, methods, and results in these Supplemental Materials for the sake of scholars who may be interested in how the strength of a culture’s social norms relates to other forms of prejudice and discrimination.

This was a 14-nation cross-cultural field experiment that aimed to test whether the cultural strength of norms predicted discriminatory behavior against people with stigmatized traits. While there have been field studies on how stigmatized individuals are treated in public places within the United States (1), this study represents the first field experiment to study cross-national differences in stigma-based discrimination.

In this study, native hypothesis-blind research assistants from 14 countries interacted with random strangers in two public contexts in which they ostensibly needed help: asking for directions to the nearest train station on the city street and asking for help picking out clothing in a retail store. As in previous research (1), we treated instances where research assistants did not receive help as cases of discrimination. Research assistants carried out their interactions between 15 and 45 times for each location in each country, and our final dataset had 768 observations.

We included one control condition and two “stigma” conditions. In a control condition, confederates wore typical clothing and no distinguishing features. In one stigma condition, confederates wore prosthetic warts on their face (“warts” condition). In the other stigma condition, they wore prosthetic tattoos across their neck, a dyed hair extension, and several facial piercings (“tattoos” condition). We designed both stigmas so that they were clearly visible from a distance. Our hypothesis was that cross-cultural variance in strength of norms would negatively predict rates of helping, such that fewer confederates with stigmatized features would receive help in tight versus loose nations.

After their interactions, the hypothesis-blind research assistants rated how much help they received. The lowest value of this response variable represented active refusal to help (e.g. verbal or physical dismissal), the middle value represented instances where the confederate was not helped, but also not refused (e.g. stopping and apologizing), and the highest value represented instances where the confederate was helped (e.g. stopping and directing the confederate to the metro station). Of the 768 interactions, research assistants were not helped in 36. Seven of these instances featured active refusal.

We conducted our analyses using multilevel logistical regression. Tattoo and warts condition were each dummy-coded and interacted separately with TL. Intercepts and slopes associated with the tattoo and warts conditions were modelled as randomly varying across nations. All analyses controlled for GNPPC.

**Strength of Norms and Discrimination.** How did TL affect the likelihood of discrimination? Tattoo condition interacted significantly with TL, *b* = -.04, *SE* = .01, *t* = -3.59, *p* = .005, level-2 pseudo *R^2^* = .01, and this effect was not moderated by the directions or store context, *b* = .0006, *SE* = .02, *t* = .05, *p* = .96, level-2 pseudo *R^2^* = .007. The results showed that in the control condition, strength of norms was unrelated to helping behavior, *b* = -.003, *SE* = .009, *t* = -.33, *p* = .75, suggesting that strength of norms is not related to overall helping behavior. But in the tattoo condition, strength of norms was negatively and significantly associated with helping behavior, *b* = -.05, *SE* = .02, *t* = -3.23, *p* = .009. In other words, research assistants wearing tattoos were helped less as a function of societal strength of norms, but research assistants without stigmatized features were helped similarly across tight and loose societies.

In this same model, a marginally significant three-level warts condition $\times$TL$\times$context interaction emerged, *b* = -.03, *SE* = .01, *t* = -1.84, *p* = .07, level-2 pseudo *R^2^* = .008. In the store condition, warts condition did not significantly interact with TL, *b* = -.004, *SE* = .01, *t* = -.35, *p* = .73. However, in the directions condition, the interaction was significant, *b* = -.03, *SE* = .01, *t* = -2.66, *p* = .01, such that research assistants in the warts condition—but not the control condition—faced greater discrimination as a function of strength of norms, *b* = -.03, *SE* = .01, *t* = -3.42, *p* = .003. This tentatively suggests that discrimination against research assistants with warts was apparent on the city streets but not in a retail store. Further exploration analysis showed that research assistants with warts were indeed helped in every single instance they entered the retail store, regardless of societal strength of norms. Differences in the strength of situations may have moderated the effect of strength of norms on discrimination. Workers in a retail store operate within a stronger situation, since they are obliged to help people even if they have physical abnormalities such as facial warts. However, people on city streets have no such obligation.

**Supplemental References**

1. Morgan WB, Walker SS, Hebl M, King EB. A field experiment: Reducing interpersonal discrimination toward pregnant job applicants. Journal of Applied Psychology, 2013; 98, 799-809.

1. Project Implicit also hosts an anti-weight IAT. However, there were major formatting changes during the course of this test that made data from different years incomparable. For this reason, we did not analyze this IAT. [↑](#footnote-ref-1)
